# Primary thymic MALT lymphoma in a patient with Sjögren’s syndrome and multiple lung cysts: a case report

**DOI:** 10.1186/s40792-019-0696-4

**Published:** 2019-09-02

**Authors:** Yusuke Hirokawa, Ryo Fujikawa, Yoshifumi Arai, Yoshiro Otsuki, Toru Nakamura

**Affiliations:** 1 Department of General Thoracic Surgery, Seirei Hamamatsu General Hospital, 2-12-12 Sumiyoshi, Nakaku, Hamamatsu City, Shizuoka 430-8558 Japan; 2 Department of Pathology, Seirei Hamamatsu General Hospital, 2-12-12 Sumiyoshi, Nakaku, Hamamatsu City, Shizuoka 430-8558 Japan

**Keywords:** Thymic MALT lymphoma, Sjögren’s syndrome, Lung cysts

## Abstract

**Background:**

Thymic mucosa-associated lymphoid tissue (MALT) lymphoma is rare and also known for its association with autoimmune diseases, especially Sjögren’s syndrome (SjS), which could affect the systemic organs, and pulmonary involvement often reveals multiple lung cysts.

**Case presentation:**

A 40-year-old woman presented with an anterior mediastinal mass and multiple lung cysts on computed tomography. We suspected thymoma concomitant with lymphangioleiomyomatosis and performed a total thymectomy and wedge resection of the lung as a surgical biopsy. The histopathological diagnosis of the mediastinal mass was a MALT lymphoma, and there were no specific findings in the lung specimen. She had a history of SjS, which had been overlooked during the initial work-up.

**Conclusions:**

A history of SjS should raise suspicion of a MALT lymphoma for the differential diagnosis of an anterior mediastinal mass. A precise history taking is crucial for the correct diagnosis, and we could have avoided a lung resection in our case.

## Background

Mucosa-associated lymphoid tissue (MALT) lymphoma is a rare form of low-grade B cell lymphomas [1]. While the stomach is the most common site, the spleen, salivary glands, skin, lungs, and orbit could also be involved [2–8], and a thymic origin is extremely rare [4, 9, 10]. Autoimmune disorders such as Sjögren’s syndrome (SjS) have been associated with the development of non-Hodgkin lymphomas (NHL) [11–13], and SjS could affect not only the lacrimal or salivary glands but also the systemic organs including the lungs, which often are revealed to have multiple thin-walled cysts [14]. Here, we report a case of primary thymic MALT lymphoma in a patient with SjS exhibiting multiple lung cysts.

## Case presentation

**Fig. 1:**
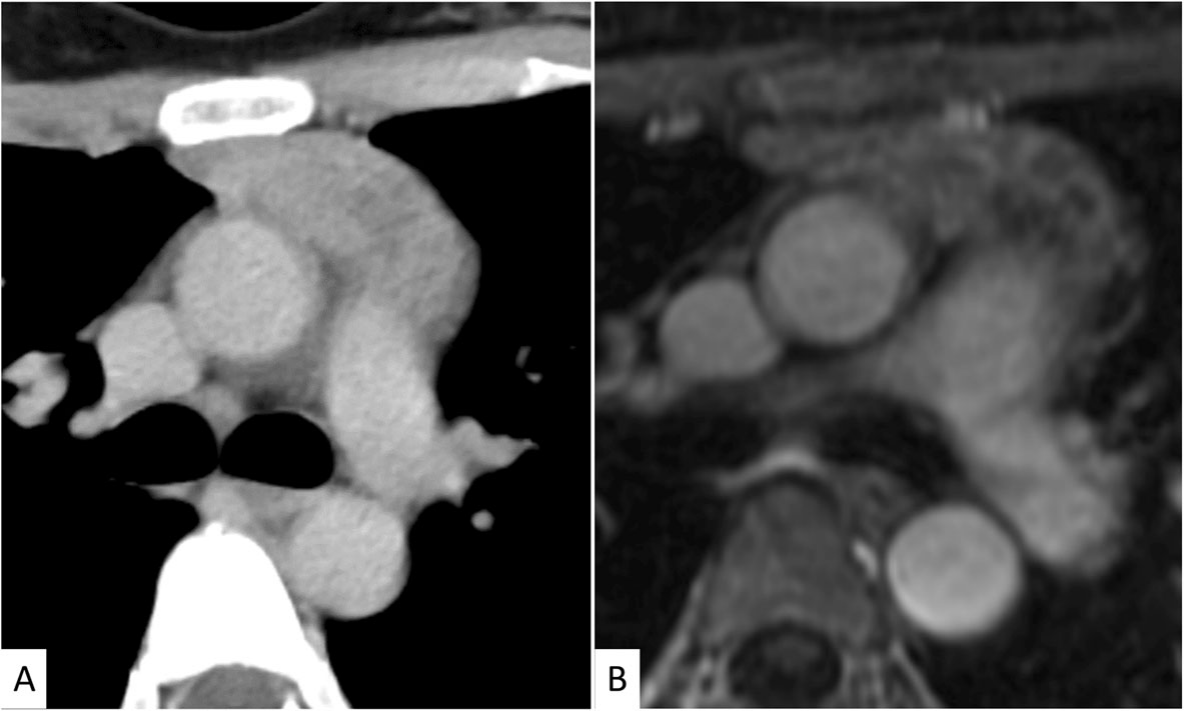
**a** Computed tomographic scan of the chest showing a well-circumscribed mass with a heterogenous concentration measuring 49 × 22 mm in the anterior mediastinum. **b** Out-of-phase dynamic magnetic resonance imaging of the mediastinum showing the multilocular mass without invasion to the surrounding parenchyma

**Fig. 2:**
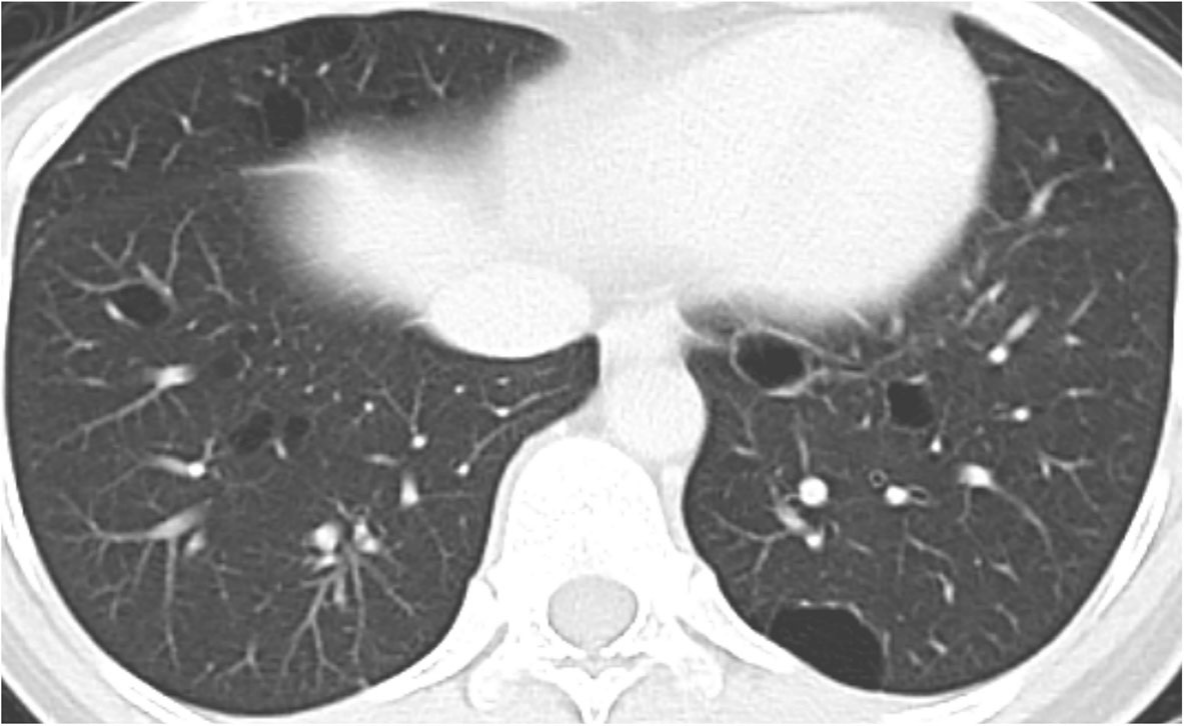
Computed tomographic scan of the chest showing multiple cysts in both lungs

**Fig. 3:**
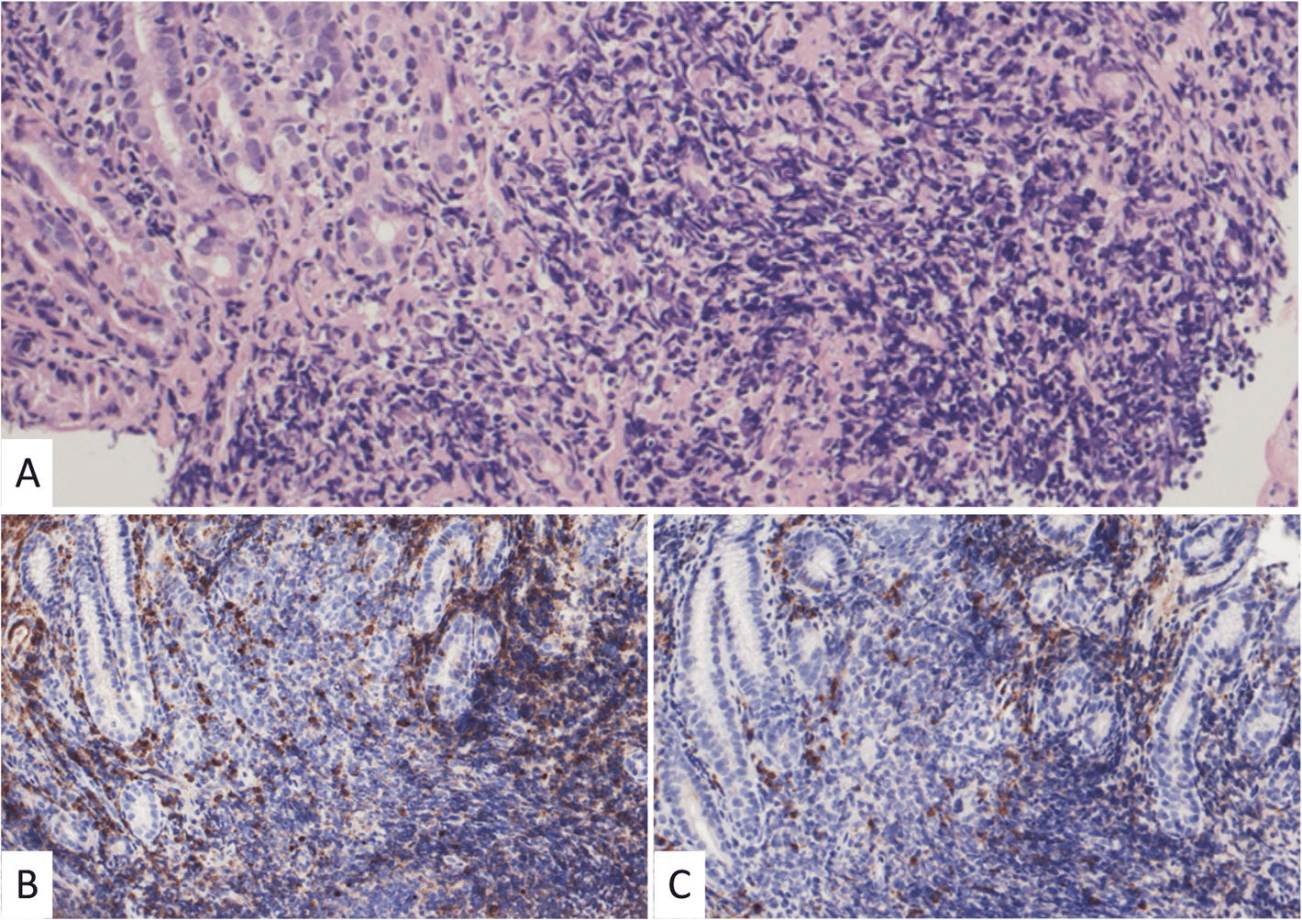
Histopathological findings of the stomach lesion showing lymphocytic infiltration without any atypical cells in the hematoxylin and eosin staining (**a**) or any light chain restriction between the kappa (**b**) and lambda (**c**) chains in the immunostaining, consistent with inflammatory changes. We used antibodies with the product number n1510 (company, DAKO; dilutions, 5 times for the kappa chains) and product number ncl-lam (company, Leica; dilutions, 200 times for the lambda chains). The positive cells are stained with a brown color

**Fig. 4:**
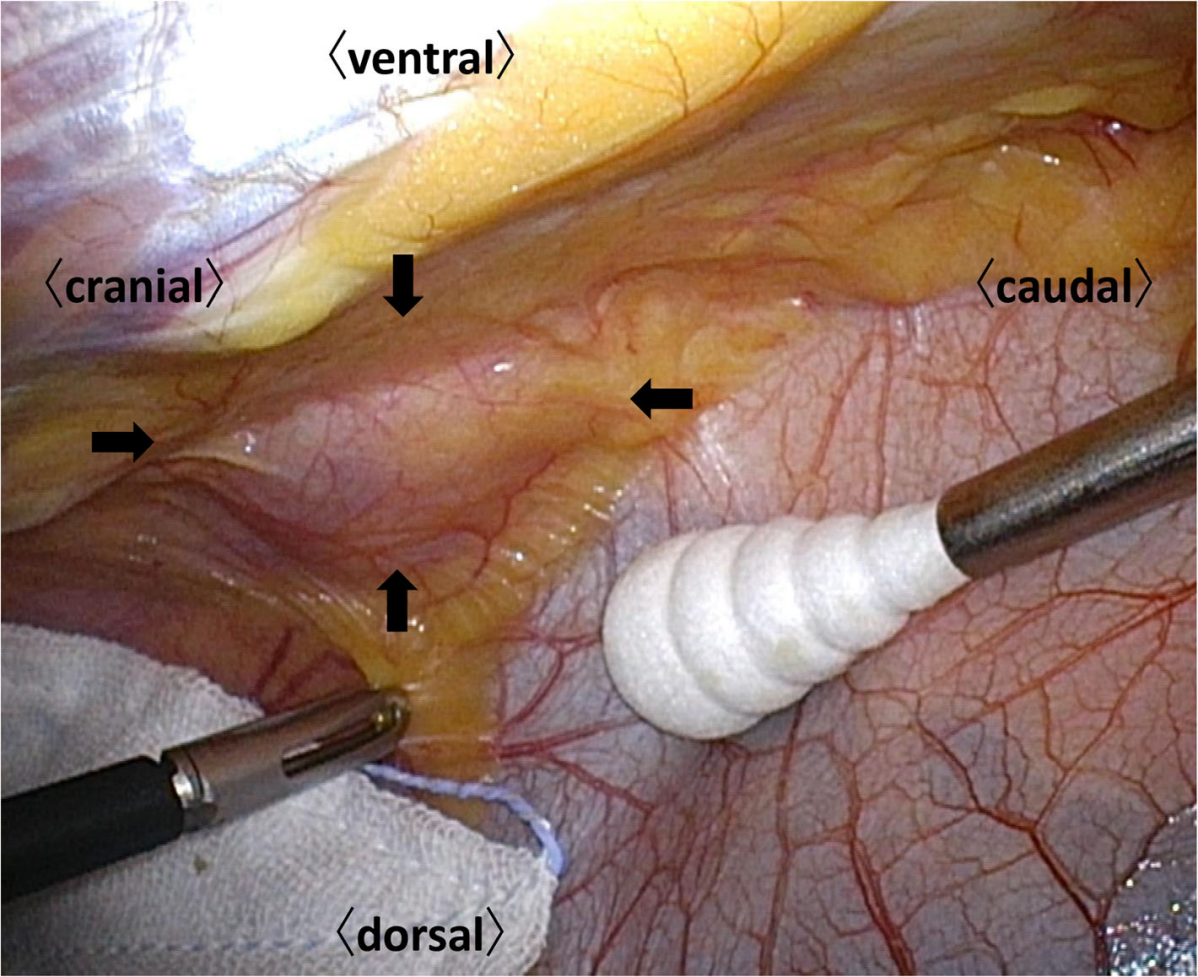
A total thymectomy was performed by a bilateral approach via a video-assisted thoracoscopic surgery. The mass is enclosed by the arrows without any adhesions to the surrounding tissue

**Fig. 5:**
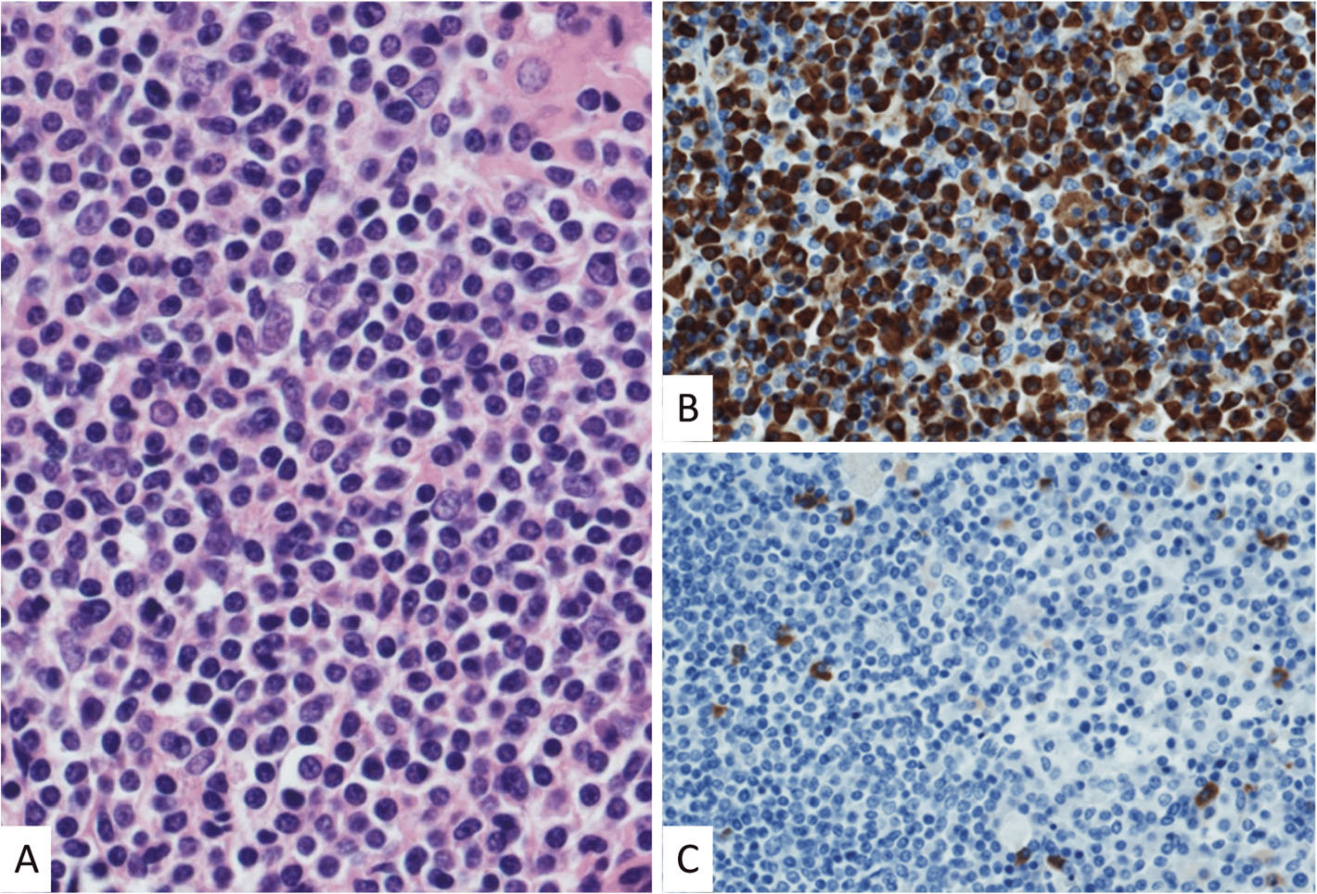
Histopathological findings of the mediastinal mass showing small- to medium-sized atypical lymphoid cells, and some of them exhibit plasmacytoid differentiation in the hematoxylin and eosin staining (**a**). A light chain restriction positive for kappa (**b**) and negative for lambda (**c**) chains is demonstrated. We used antibodies with the product number N1510 (company, DAKO; dilutions, 5 times for the kappa chains) and product number NCL-LAM (company, Leica; dilutions, 200 times for the lambda chains). The positive cells are stained with a brown color

An asymptomatic 40-year-old woman presented with gastric wall thickening detected by screening with an upper gastrointestinal series and underwent contrast-enhanced computed tomography (CT), which also revealed an anterior mediastinal mass. She had been diagnosed with asymptomatic SjS at the age of 35, which was overlooked during the initial work-up. She had no medication or smoking history. She had a family history of breast cancer in her mother’s side and rheumatoid arthritis in her father’s side. The laboratory data showed nothing but hypergammaglobulinemia (IgG, 2750 mg/dL; IgA, 625 mg/dL; IgM, 241 mg/dL), and the serum soluble interleukin-2 receptor level was also within the normal limits (362 U/mL). A chest radiograph revealed no abnormalities. An abdominal CT revealed a localized wall thickness measuring 22 mm with enhancement in the middle part of the gastric body on the greater curvature. A well-circumscribed mass with a heterogenous concentration measuring 49 × 22 mm in the anterior mediastinum (Fig. 1a) without any distant metastases and multiple cysts in both lungs (Fig. 2) were also demonstrated. Magnetic resonance imaging revealed a multilocular mediastinal mass without invasion to the surrounding parenchyma (Fig. 1b). ^18^Fluoro-2-deoxyglucose positron emission tomography (FDG-PET) was not performed before the surgery. Suspecting the gastric wall thickness suggested a gastrointestinal stromal tumor or MALT lymphoma, we performed an endoscopic incisional biopsy. The pathological findings showed lymphocytic infiltration without any atypical cells (Fig. 3a) or a light chain restriction between the kappa (Fig. 3b) and lambda (Fig. 3c) chains on immunostaining, consistent with inflammatory changes. We successfully performed a total thymectomy, due to suspecting an anterior mediastinal mass as a thymoma, by a bilateral approach via video-assisted thoracoscopic surgery with carbon dioxide insufflation in a supine position. There were no adhesions around the mass, and it was removed from the surrounding organs without any surgical difficulty (Fig. 4). We also performed a wedge resection of the right upper lobe of the lung as a surgical biopsy to rule out lymphangioleiomyomatosis (LAM). The operation time was 187 min with 2 g of total blood loss. The cut surface of the mediastinal tumor exhibited a grayish-white solid mass with multiple cysts. Histopathologically, there was an infiltration of numerous lymphoid cells with lymphoid follicles. Small- to medium-sized atypical lymphoid cells were observed, and some of them exhibited plasmacytoid differentiation (Fig. 5a). Cytokeratin immunostaining revealed a lymphoepithelial lesion, consistent with infiltration of lymphoid cells into the epithelium. These cells were positive for CD20 and negative for CD3, CD5, and CD10. A light chain restriction positive for kappa (Fig. 5b) and negative for lambda (Fig. 5c) chains was demonstrated. From these findings, we diagnosed the mediastinal mass as a MALT lymphoma. A lung cyst was found to be an emphysematous bulla without any specific histological findings suggesting LAM. The postoperative course was uneventful, and she was discharged home on postoperative day 2. FDG-PET revealed no abnormal FDG uptakes in any organs 1 month later, and she is currently disease-free at 9 months after surgery.

## Comment

MALT lymphomas are low-grade B cell lymphomas that occur in a marginal zone of MALT [1]. The stomach is the most common site and thymic involvement is extremely rare [4, 9, 10]. Now, it is known that chronic antigenic stimulation is associated with an increased risk of a MALT lymphoma, and accumulating evidence has proven the link between a primary MALT lymphoma of the stomach and a *Helicobacter pylori* infection [15–17], small intestine with *Campylobacter jejuni*, skin with *Borrelia burgdorferi*, ocular adnexa with *Chlamydia psittaci* [18, 19], and thyroid with Hashimoto’s thyroiditis [20, 21]. Primary thymic MALT lymphomas are associated with autoimmune diseases, especially SjS, which could affect the systemic organs, and pulmonary involvement often reveals multiple lung cysts accounting for 30% [22–26]. While inflammatory cell infiltration into the bronchiolar wall might cause an airway restriction with a check valve resulting in a cyst formation, the detailed mechanism has not yet been elucidated [14, 27, 28]. There were no specific pathological findings indicating the participation of SjS in our case. Primary thymic MALT lymphomas are prevalent in middle-aged Asian women and often reveal a multilocular appearance radiographically [10, 29–31]. These findings suggest that our case had typical epidemiologic and radiological characteristics of both a thymic MALT lymphoma and pulmonary involvement with SjS. However, we could not link the MALT lymphoma to the lung cysts because we overlooked her medical history of SjS. We considered that the precise recognition of the history might have led us to the correct diagnosis before the surgery because SjS was accountable for both the multiple lung cysts and NHL [11–14]. From a diagnostic point of view, a surgical resection is recommended to obtain an adequate volume of the tissue for a histological examination of the MALT lymphoma [32]. We considered that a total thymectomy in our case was feasible and also as a therapeutic modality. However, we could have avoided a pulmonary resection as a surgical biopsy. We should have paid much more attention to a careful history taking in detail in addition to the radiological evaluation.

## Conclusions

An anterior mediastinal mass with a multilocular appearance and thin-walled pulmonary cysts concomitant with a history of SjS should raise a suspicion of a primary thymic MALT lymphoma as the most likely differential diagnosis. A precise history taking is crucial to improve the quality of the diagnostic work-up and also to avoid useless surgery.

## Data Availability

Not applicable.

## Abbreviations

CT: Computed tomography
FDG-PET: ^18^Fluoro-2-deoxyglucose positron emission tomography
IgA: Immunoglobulin A
IgG: Immunoglobulin G
IgM: Immunoglobulin M
LAM: Lymphangioleiomyomatosis
MALT: Mucosa-associated lymphoid tissue
NHL: Non-Hodgkin lymphoma
SjS: Sjögren’s syndrome
